# Do environmentally induced DNA variations mediate adaptation in *Aspergillus flavus* exposed to chromium stress in tannery sludge?

**DOI:** 10.1186/s12864-018-5244-2

**Published:** 2018-12-04

**Authors:** Akanksha Jaiswar, Deepti Varshney, Alok Adholeya, Pushplata Prasad

**Affiliations:** TERI-Deakin Nanobiotechnology Centre, TERI Gram, The Energy and Resources Institute, Gual Pahari, Gurgaon Faridabad Road, Gurgaon, Haryana 122 001 India

**Keywords:** Non synonymous SNPs (nsSNPs), Mutation, Protein structure and function, Protein-ligand interaction, Adaptation

## Abstract

**Background:**

Environmental stress induced genetic polymorphisms have been suggested to arbitrate functional modifications influencing adaptations in microbes. The relationship between the genetic processes and concomitant functional adaptation can now be investigated at a genomic scale with the help of next generation sequencing (NGS) technologies. Using a NGS approach we identified genetic variations putatively underlying chromium tolerance in a strain of *Aspergillus flavus* isolated from a tannery sludge. Correlation of nsSNPs in the candidate genes (*n* = 493) were investigated for their influence on protein structure and possible function. Whole genome sequencing of chromium tolerant *A. flavus* strain (TERIBR1) was done (Illumina HiSeq2000). The alignment of quality trimmed data of TERIBR1 with reference NRRL3357 (accession number EQ963472) strain was performed using Bowtie2 version 2.2.8. SNP with a minimum read depth of 5 and not in vicinity (10 bp) of INDEL were filtered. Candidate genes conferring chromium resistance were selected and SNPs were identified. Protein structure modeling and interpretation for protein-ligand (CrO_4_^− 2^) docking for selected proteins harbouring non-synonymous substitutions were done using Phyre2 and PatchDock programs.

**Results:**

High rate of nsSNPs (approximately 11/kb) occurred in selected candidate genes for chromium tolerance. Of the 16 candidate genes selected for studying effect of nsSNPs on protein structure and protein-ligand interaction, four proteins belonging to the Major Facilitator Superfamily (MFS) and recG protein families showed significant interaction with chromium ion only in the chromium tolerant *A. flavus* strain TERIBR1.

**Conclusions:**

Presence of nsSNPs and subsequent amino-acid alterations evidently influenced the 3D structures of the candidate proteins, which could have led to improved interaction with (CrO_4_^− 2^) ion. Such structural modifications might have enhanced chromium efflux efficiency of *A. flavus* (TERIBR1) and thereby offered the adaptation benefits in counteracting chromate stress. Our findings are of fundamental importance to the field of heavy-metal bio-remediation.

**Electronic supplementary material:**

The online version of this article (10.1186/s12864-018-5244-2) contains supplementary material, which is available to authorized users.

## Background

Bioremediation of heavy metals by microbial cells has been recognized as a potential alternative to the existing physico-chemical technologies for recovery of heavy metals from industrial effluents [1]. Metal uptake in microorganisms takes place either actively (bioaccumulation) or passively (biosorption) [2–6]. Several species of bacteria and fungi have been identified for their bioaccumulation or absorption potentials and reduced cost and toxicity achieved by microbial bioremediation approach are appreciated over the conventional methods [7]. Various bacterial species detoxify chromium by periplasmic absorption, intracellular bioaccumulation and biotransformation through direct enzymatic reaction or indirectly with metabolites. Filamentous fungi have been identified as a potential biomass for removal of heavy metals from solutions and species of *Aspergillus*, *Rhizopus* and *Penicillium* are reported useful in biological treatment of the sludge [8–11], Several reports support the prominent ability of *Aspergillus flavus* in detoxification of chromium and other heavy metals [12]. However, the molecular mechanisms underlying heavy metal detoxification in fungi are largely unknown. Understanding the genes and pathways involved in metal accumulation/tolerance in fungi has several biotechnological implications for bioremediation of heavy metal-contaminated sites.

The extensive use of chromium in diverse industrial processes has made it a significant environmental contaminant. Chromium is a Class A human carcinogen [13, 14] and exists in eleven valence states (from −IV to +VI), among which Cr (III) and Cr (VI) are the most stable forms in the environment. Due to high water solubility Cr (VI) is 100-folds more toxic over Cr (III). As per the United States Environmental Protection Agency (US EPA) the maximum contaminant level for Cr (VI) and total chromium content in domestic water supplies is 0.05 and 2 mg/l respectively [15]. Cr (VI) actively crosses biological membranes [16] and generates active intermediates Cr (V) and/or Cr (IV), free radicals, and Cr (III). Cellular accumulation of Cr (III) causes damage to DNA and alters the structure and activity of proteins [17, 18]. The existing physico-chemical processes for treating chromium-contaminated water bodies *include* precipitation, ion exchange, reverse osmosis, evaporation and electro dialysis, which are reported to display poor efficiency [14, 19–24].

For survival in Cr (VI) contaminated environments, microorganisms must develop efficient systems to detoxify the effects of chromium. These mechanisms involve detoxification or repair strategies such as Cr (VI) efflux pumps, Cr (VI) reduction to Cr (III), and activation of enzymes involved in the detoxifying processes, repair of DNA lesions, sulfur metabolism, and iron homeostasis [16, 18, 25]. Additionally, alterations in gene function due to mutation have been suggested to support survival under chromium toxic conditions [26]. Biotransformation and biosorption are suggested as the putative fungal processes that help them transform or adsorb heavy metals [27]. The fungal cell walls predominantly consist of chitins, glucans, mannans and proteins in addition to other polysaccharides, lipids and pigments [28, 29]. The functional groups on these structural components enable binding of metal ions on the fungal cell walls [30]. Uptake and reduction of hexavalent chromium has been suggested as the mechanisms for chromium tolerance in *Aspergillus sp*. [27, 31].

Information on genes supporting survival under environmental stress in bacterial system has been recently curated in BacMet database (http://bacmet.biomedicine.gu.se) which primarily contains several experimentally verified Chromate ion transporter (CHR) genes [32] responsible for chromium efflux, transport or binding, and other enzymes involved in chromium uptake. However, very less knowledge is available on genetic mechanisms responsible for chromium tolerance in fungi. In the *Neurospora crassa* strain 74-A, chr-1 gene that encodes a putative CHR-1 protein and belongs to the CHR superfamily was identified [33]. However, contrary to the bacterial ChrA (chromate transport protein) homologues that confer chromate resistance by exporting chromate ions from the cell’s cytoplasm, the experimental data suggested that the *N. crassa* CHR-1 protein functions as a transporter that takes up chromate [34]. The presence of CHR-1 protein was reported to cause chromate sensitivity and chromium accumulation in *N. crassa*.

Experimental evidences in a recent study suggested that environmental stress could induce adaptation in a wide range of micro-organisms by extensive positive pleiotropy in a manner that multiple beneficial mutations dramatically enhance numerous fitness components simultaneously [35]. Environmentally induced mutations and polymorphisms in DNA and subsequently the alteration in proteins are hypothesized to offer a significant evolutionary advantage by enabling faster adaptation to toxic conditions [36]. We identified a high chromium tolerant *Aspergillus flavus* strain (TERIBR1) from a tannery sludge in Kanpur, Uttar Pradesh, India. TERIBR1 showed accumulation of Cr (III) in its biomass while growing in Cr containing media. It showed no toxic effect of Cr (VI) up to 250 mg/l. In order to identify the genetic factors underlying chromium tolerance in TERIBR1, we investigated effects of nonsynonymous variations (nsSNPs) in candidate genes on protein structure and their interaction with chromate ion.

Our study comprises whole genome sequencing of *A. flavus* strain TERIBR1 followed by single nucleotide polymorphism (SNPs) analysis in candidate genes for chromium-resistance. Protein modeling for candidate genes with nsSNPs was done and interactions between modeled proteins and the ligand (CrO_4_^− 2^) were assessed by protein-ligand docking. For all comparative genomics and genetics analyses the *A. flavus* strain TERIBR1 was considered as the “test” and previously sequenced strain NRRL3357 as the “reference” type.

## Materials and methods

### Fungal strain and DNA extraction

The protocol followed for isolation and characterization of fungi from a tannery sludge is previously described [37]. Briefly, the Cr-resistant fungi were isolated from a tannery sludge [containing 250 mg/l of Cr (III)] through an enrichment culture technique. The sludge sample was collected from a tannery waste disposal site in Kanpur, India. Pure culture of the isolated *A. flavus* strain (TERIBR1) was grown in potato dextrose broth (PDB) at 28 °C in a shaking incubator (100 rpm) for 72 h in dark condition. After incubation, culture was centrifuged at 5000 g for 10 min at room temperature. The pellet was washed thrice with sterile distilled water to remove any media components and was further used for DNA extraction. Genomic DNA was extracted using the DNeasy plant mini kit (QIAGEN, USA), according to the manufacturer’s instructions. Genetic characterization of isolated fungi was done using universal fungal ITS (nuclear ribosomal internal transcribed spacer) primer set [ITS1: 5’ TCCGTAGGTGAACCTGCGG, 3′ and ITS4: 5’ TCCTCCGCTTATTGATATGC 3′; [38] that amplified the ITS1, 5.8S and ITS2 regions of the nuclear ribosomal RNA genes.

### Growth kinetics and sensitivity to Cr (VI)

The effect of different concentrations of chromium [Cr (VI)], 0 mg/l, 100 mg/l and 250 mg/l, on the growth of *A. flavus* strains TERIBR1 and NRRL3357 was compared. The strains were grown in PDB and mycelial biomass (dry weight) was measured at different time periods (0, 1, 2, 3, 4 and 5 days).

### Genome sequencing and assembly

Genome sequencing was performed at MOgene LC, USA, using next generation sequencing technology Illumina as reported previously [39]. Two paired end libraries (insert sizes 180 bp and 500 bp) and one mate pair library (5 kb) were constructed. DNA libraries were purified using AMPure XP beads. KAPA was done to quantify the libraries, which were then normalized and pooled at 4 nM concentration.

A total of 8 GB raw data was subjected to adaptor- and quality-based trimming. Quality-passed data was assembled using the de novo genome assembler AllpathsLG [40]. Reads with overlaps were first combined to form contigs. The reads were mapped back to contigs. With paired-end reads, contigs from the same transcript, as well as the distances between these contigs, were detected. In order to generate scaffolds, contigs were connected using “N” to represent unknown sequences between two contigs. Mate-pair reads were used for gap filling of scaffolds in order to get sequences with minimal N’s and the longest length. The whole genome project has been deposited at https://submit.ncbi.nlm.nih.gov/subs/wgs/under Bioproject PRJNA362980.

Structural and functional annotation of *A. flavus* TERIBR1 genome was done using MAKER [41] pipeline, InterProScan [42] and nrBlast [39] as described previously.

### Identification of single nucleotide polymorphisms (SNPs)

Genome and protein sequences for reference genome were retrieved from the *Aspergillus flavus* Database (http://fungidb.org/fungidb/app/record/organism/aflaNRRL3357). The alignment of quality trimmed data of TERIBR1 with NRRL3357 (assembly) was performed using Bowtie2 version 2.2.8 [43]. Samtools [http://samtools.sourceforge.net/] was used for SNP identification.

### SNP analysis in candidate genes for chromium resistance

Genes conferring chromium resistance in bacterial system were selected from BacMet database [32]. BacMet is freely available antibacterial biocide and metal resistance genes database for bacteria. InterProScan analysis [42] was performed to identify *A. flavus* genes harbouring atleast one IPR domains that are present in the chromium resistance genes documented in the BacMet database. SNPs were identified in the selected candidate genes using variant calling format (VCF) file and Blastn tool. SNPs were further annotated as synonymous or non-synonymous (nsSNPs) using an in-house perl script.

### Protein structure modeling

Protein modeling was done by fold recognition methods through Phyre2 server [44]. The amino acid sequences of candidate genes in both the reference (NRRL3357) and the test strains were modeled. The top model with highest confidence and coverage was selected for each protein. The predicted confidence score and coverage for all the final structures were recorded. To assess the reliability of all the predicted models, structural analysis and verification was exercised**.** The selected models were validated using the PROCHECK [45] and ERRAT [46] to estimate the stereo chemical figures, geometry, and hydrogen bonding energy, torsion angles and error rate of the predicted structures. In addition, energy minimization was performed with in vacuo GROMOS96 43B1 parameters set using GROMOS96 implementation in Swiss-Pdb Viewer [47]. The energy optimized protein structures were used for protein-small ligand docking.

### Prediction of ligand binding sites

Prior to docking, a web based approach 3DLigandSite [48] was used to predict the ligand binding sites. 3DLigandSite utilizes protein-structure prediction to provide structural models for proteins that have not been solved. Ligands bound to structures similar to the query are superimposed onto the model and used to predict the binding site.

### Protein- ligand docking

In order to investigate protein–ligand interactions, proteins were docked with the chromate ion (CrO_4_^− 2^) through a rigid docking protocol using PatchDock (http://bioinfo3d.cs.tau.ac.il/PatchDock/) [49, 50] which docks the ligand with the protein based on structure complementarity. Also, binding sites predicted by 3DLigandSite in the receptor/proteins were specified and uploaded in PatchDock analysis. The protein-ligand interactions were interpreted based on Atomic Contact Energy (ACE) and docking score. The pdb file of chromate ion was downloaded from the RCSB PDB (research collaborator fo structural Bioinformatics protein data bank) site [51]. The PDB structures of target proteins and protein-ligand interaction were visualized using the PyMOL [52].

## Results

### Growth kinetics and sensitivity to Cr (VI)

**Fig. 1:**
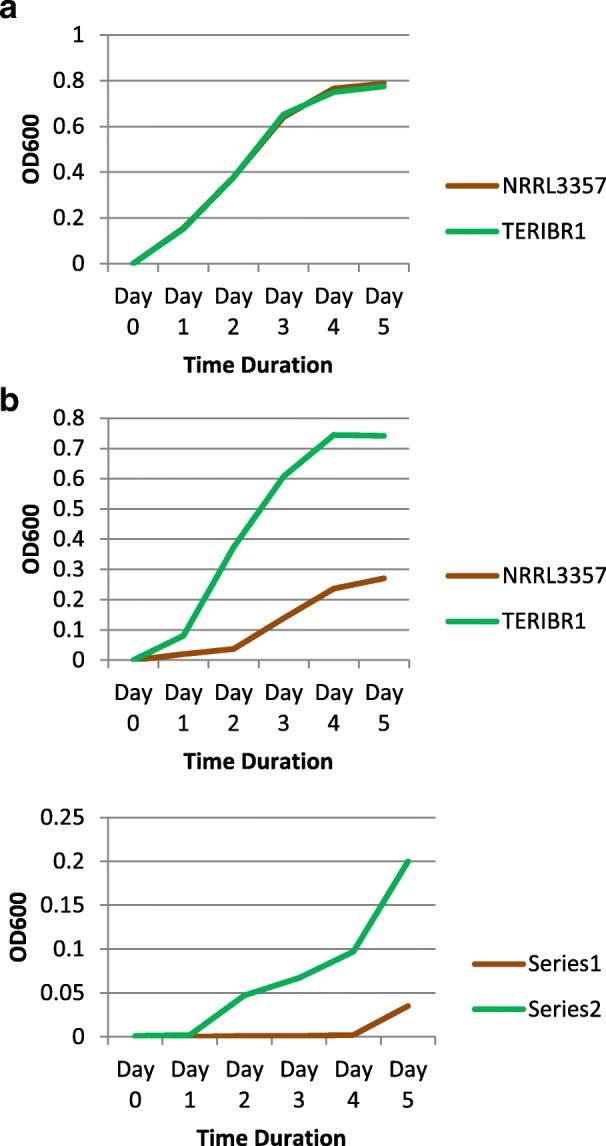
Chromium [Cr (VI)] dose response exhibited by TERIBR1 and NRRL3357 strains of *A. flavus.* Chromium dose/growth response (measured by dry weight) exhibited by TERIBR1 and NRRL3357 strains of *A. flavus* grown up to 5 days in potato dextrose broth supplemented with Cr (VI): (**a**) 0 mg/l, (**b**) 100 mg/l and 250 mg/l

Dry weight of fungal biomass was recorded at different time periods (from 1 to 5 days) for both the strains under the conditions mentioned above. No significant difference in growth was observed between the two strains under the control condition (Fig. 1). However, stark difference in the mycelial biomass (dry weight) between the reference strain (NRRL3357) and the test strain (TERIBR1) was observed when potato dextrose broth was amended with chromium 100 mg/l and 250 mg/l. Growth kinetics of the TERIBR1 strain at chromium concentration of 100 mg/l were similar to that observed under control condition (no chromium). The reference strain exhibited delayed growth response with concomitant decrease in biomass in comparison to the test strain at different time intervals (between day 1 and day 5) when the growth media was amended with chromium at concentrations of 100 mg/l and 250 mg/l.

### Global genome structure

The genome of *A. flavus* strain TERIBR1 was sequenced to 200x coverage and reads were assembled into 322 scaffolds. The sum of the scaffolds length is equal to 38.2 Mb. The three largest scaffolds are 2.76 kb, 2.64 kb, and 2.50 kb in size. The MAKER annotation pipeline predicted 13,587 protein coding genes as compared to 13,659 in NRRL3357. Gain or loss of unique genes, DNA duplication, gene family expansion, and translocation of transposon-like elements are often observed between different isolates of a fungal species [53]. This may suggest that some of the genes present in NRRL3357 could have been lost in TERIBR1, possibly during environmental adaptations.

### Identification of candidate genes in *A. flavus*

No homologue of CHR-1 protein (XP_961667.3) coded by *N. crassa* was identified in both the *A. flavus* strains included in this study. A total of 34 InterProScan domains coding for transporter or regulator proteins responsible for chromium bio-accumulation or tolerance in bacteria were reported in the BacMet database. nrBlast was performed to identify genes containing at least one IPR domain associated with chromium tolerance in the genome of *A.*

*Flavus* strain TERIBR1, NRRL3357 (http://fungidb.org/fungidb/app/record/organism/aflaNRRL3357) and AF70 (https://www.ncbi.nlm.nih.gov/assembly/GCA_000952835.1). 23/34 bacterial IPR domains were not found in any of the three strains of *A. flavus*. A total of 493 candidate genes was identified to harbor one or more IPR domains of interest in TERIBR1(Table 1). IPR domains mdrL/yfmO (IPR011701; *n* = 334), recG (IPR001650; *n* = 71), ruvB (IPR003959; *n* = 45) and recG (IPR011545; *n* = 44) were among the maximally present protein domains related to chromium resistance.

**Table 1:** Distribution of IPR domains important in chromium bio-accumulation in *A. flavus* strains TERIBR1, NRRL3357 & AF70

Gene Family (BacMet db)	Description	Interproscan Domain	# of Genes containing IPR domains of interest		
			NRRL3357	TERIBR1	AF70
Chromate ion transporter (CHR) family (chrA)	Efflux	*IPR003370*	1	1	2
Rhodanese family (chrE)	Enzyme	*IPR001763*	9	6	10
NADH_dh2 family (chrR)	Enzyme	*IPR005025*	4	4	4
		*IPR000415*	0	3	0
MFS superfamily (mdrL/yfmO)	Efflux	*IPR011701*	374	334	394
Contains 1 DEAD/DEAH box helicase domain (recG)	Enzyme	*IPR011545*	43	44	42
		*IPR001650*	74	71	80
		*IPR004365*	2	5	4
RuvB family (ruvB)	Enzyme	*IPR003959*	47	45	48
		*IPR012301*	2	2	2

### Identification of single nucleotide polymorphisms (SNPs)

The read alignment rate of TERIBR1 with NRRL3357 (assembly) was 78.62% (29,001,807 / 36,890,268) of which 78.23% (22,681,743) were uniquely mapped reads. A total of 201,145 SNPs (read depth > 5) was identified at a frequency of ~ 5 SNPs per Kb of the TERIBR1 genome. SNP mapping in *n* = 493 candidate genes, homologous among *A. flavus* NRRL3357 and TERIBR1 isolates was done using Samtools. No SNP was identified in 325/493 genes. SNPs identified in the remaining *n* = 168 genes were annotated as synonymous or non-synonymous (Additional file 1: Table S1). 28/168 candidate genes contained only synonymous polymorphisms whereas 16/168 candidate genes, belonging to MFS (*n* = 12), recG (*n* = 3) and chrE (*n* = 1) protein families, showed higher rate of nsSNP as compared to other candidate genes (Additional file 2: Table S2).

### Protein- ligand docking

**Fig. 2:**
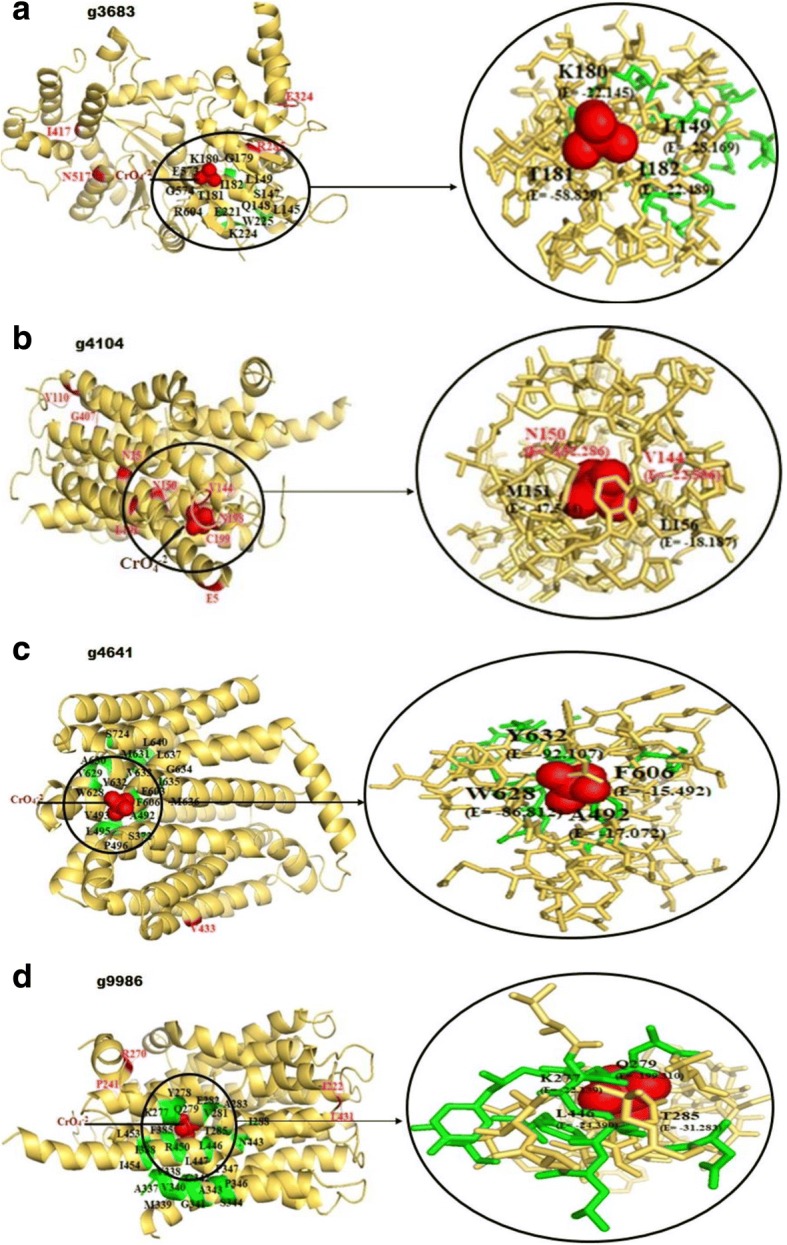
Protein-chromate ion interaction observed with four MFS transporter proteins of *A. flavus* strain TERIBR1. Docking of chromate ion with MFS transporter proteins in occluded conformation. The chromate ion is depicted as a sphere model. The amino acids of the interacting protein showing negative energy are depicted as bright orange sticks and the interacting binding sites as green sticks. Presence of nsSNPs in the protein sequence is shown in magenta. Amino-acids present in the close vicinity of the binding sites are marked in black (sSNP) and magenta (nsSNP). Figure was produced using the PyMOL Molecular Graphics System

For studying protein-chromate ion interaction, we predicted tertiary protein structures of homologous pairs of the 16 highly polymorphic proteins (Additional file 2: Table S2) using Phyre2 server (Additional file 3: Table S3). Prediction for Cr binding sites in the target proteins was done by 3DLigandSite (Table 2). Strength of protein-ligand interaction was measured based on the atomic contact energy (ACE) in the PatchDock score (Table 3). Also change in free energy (ΔG) of the amino acid residues present in the predicted binding and ligand docking sites was recorded (Fig. 2). Structures of 8 proteins in both the reference and test strains did not show any possible interaction between the ligand and the target proteins. Ligand docking was observed in both the strains for four proteins (g8975, g685, g6212, g9525; Additional file 4: Figure S1). Binding residues that showed a drop in free energy on chromate docking in PatchDock analysis are depicted on the 3D structures of these four proteins (Additional file 4: Figure S1).

**Table 2:** Prediction of binding site and protein – ligand interaction using 3DLigandSite and PatchDock softwares respectively

Protein ID		nsSNP	Predicted binding sites		Docking status and residues in recognition cavity		# nsSNP	Gene Family
NRRL3357	TERI BRI		NRRL 3357	TERI BR1	NRRL3357	TERIBR1		
AFL2G_00299	g652	A346D, D351N, M389 T	A261, G262, I263	No B.S.	N/A		≤2	mdrL/ yfmO
AFL2G_04853	g9548	P254L, K261I, K263E, A262D, M34 T	T67, F68, V69, S70, P71, L72, A73, S74, S75, L104, Y107, V108, P111, G161, C164, L165, W188, P192, Y280, L283, Y284, T288, Y393, T416, A417, S420, L421, V422, A424, L425, L426	Y122, W203, P207, Y319, L322, Y323	N/A		2 to 5	mdrL/ yfmO
AFL2G_04391	g8975	P341A, D349E, H356Y, P373L, P374L	Q119, F240, H403, T404, N405, V407, Q408, L454, F477, S481, Y485, V508, L511, Q512, V514, S515, R516, F518, V519, L520, P521, S524	Q115, F240, N405, A406, Q408, T409, L454, F477, S481, Y485, V508, L511, Q512, V514, S515, R516, F518, V519, L520, P521, S524, R552	Dock		2 to 5	mdrL/ yfmO
AFL2G_02473	g5755	K53 N, N59D, K213 M, V293I, K340R	H97, W124, I125, L126, V127, M128, F129, F130, A131, L132, N133, I134, D135, I183, G184, P185, D186, R187, W188, I189, P190, I191, Q192, I193, I194, L195, S197, F226, D229, V253, S257, A288, S291, I292, G295, F296, S298, F299, L302	W115, I116, L117, V118, M119, F120, A122, I174, G175, P176, D177, R178, W179, I180, P181, I182, Q183, I184, I185, L186, F217, S282, G286, S289, F290, L293, V294	N/A		2 to 5	mdrL/ yfmO
AFL2G_00264	g685	S126G, T139A, G179E, Y112F	T334, L335, G400, K401, S402, L403, E461, H465, F680, G681, R711	T321, L322, M386, G387, K388, S389, L390, E442, H446, F726, G727, R757	Dock		2 to 5	recG
AFL2G_05826	g6641	P307L, Q11P, V19G, E102D, V126A, A129V	No B.S.	S270, M273, I274, Q396	N/A		> 5	mdrL/ yfmO
AFL2G_09247	g6212	R471H, R437Q, S837P, L1229 V, V192I, L233S	K272, L273, L274, Q277, G309, L310, G311, K312, T313, V314, E380, K384, L919, G920, L921, N922, R947, R950, L951	L544, V546, K547, L548, L549, Q552, G584, L585, G586, K587, T588, V589, E655, K659, L1194, G1195, N1197, R1222, R1225, L1226	Dock		> 5	recG
AFL2G_08767	g9986	F222I, A244P, Q270P, G340R, A342G, F431 L, S472I	W290, L291, Y292, L294, M295, I353, L354, V355, M356, H357, L358, W359, T360, P362, P363, F401, I404, Y455, M458, N459, L462, T465, R466	**K277,** Y278, **Q279,** V281, E282, A283, **T285,** I288, A337, V338, M339, V340, G341, G342, A343, S344, P346, P347, F385, I388, N443, **L446,** L447, R450, L453, I454	N/A	Dock **K277, Q279, T285, L446**	> 5	mdrL/ yfmO
AFL2G_05032	g9401	N373D, S445 N, E503G, S535 L, V572G, F592Y, K610E, I50M	H614, H616, L666, H668, H670	C97, A98, F100, L101, Y104, I107, M159, A160, I161, I162, Y164, S165, A168, I169, F198, A202, V205, S257, T260, H261, A264, N267, K268	N/A		> 5	mdrL/ yfmO
AFL2G_06586	g3683	S517 N, Q324E, L899S, S57 L, D63G, T18I, K285R	Q193, L194, K195, Q198, M221, G222, L223, G224, K225, T226, I227, E266, I643	L145, S147, Q148, **L149,** G179, **K180, T181, I182,** E221, K224, W225, E573, G574, R604	N/A	Dock **L149, K180, T181, I182**	> 5	recG
AFL2G_11779	g4359	G294D, K360E, V388I, F393 L, L468P, Q66H, R19K, V637I, A646T	L582, V586, M589, N590, M593, A621, Y623, L631, H632, A635, H636, H640, W647, I659	R34, T36, A94, V95, Y100, S101, A178, I206, P207, L208, A209, V211	N/A		> 5	chrE
AFL2G_09661	g4104	R80M, L110 V, **V144 L, N150S,** F191 L, G198R, Y199C, E407G, G5E, I25N	No B.S.		N/A	Dock **V144 L, N150S**	> 5	mdrL/ yfmO
AFL2G_04878	g9525	G210S, C363W, I368S, H438Y, M484, P106A, V131I, V146A, N156S, F163 L, L182F, Q59H	S63, I66, F92	S141, I144, F170	Dock		> 5	mdrL/ yfmO
AFL2G_00229	g712	R163L, G180C, S215C, S220Y, A226P, V693I, S765 N, F834I, Q854H, C938S, V121A	G570, A571, N572, S573, G574, L575, V595, R596, S597, K600, L624, D625, M626, L627, N652, A653, G654, I655, V673, V704, G705, S706, Y745, K749, P780, G781, P782, T783, S785, G786, L787	G666, A667, N668, S669, G670, L671, V691, R692, K696, L720, D721, M722, L723, N748, A749, G750, I751, V769, V800, G801, S802, Y841, K845, P876, G877, P878, T879, S881, G882, L883	N/A		> 5	mdrL/ yfmO
AFL2G_04255	g9088	L52 V, S101G, A212T, F214 L, T217A, S237 L, A250V, P252L, P271S, M280 T, K292 N, S297R, V303I	A104, L105, P108, S110, L138, I139, V141, G142, M165, M169, A226, I256, F338, L341, N342, M367, Y477, G481, L483	A195, P198	N/A		> 5	mdrL/ yfmO
AFL2G_11442	g4641	M254I, P321T, I433V, D661G, P675Q, F682 L, D110N, A111V, I114V, K143 T, T3A, H13Q, T24A, C46S, K826R	A120, F121, V122, V123, S124, A125, A126, S127, S128, L156, F159, A160, S163, M187, P216, L217, Y240, S244, Y355, F359, D363, T513, V514, Y517, C518, A519, G521, G522, M523	S372, **A492,** V493, L494, P496, F603, **F606, W628,** V629, A630, M631, **Y632,** V633, G634, I635, M636, L637, L640, S724	N/A	Dock **Y632, F606, W628, A492**	> 5	mdrL/ yfmO

SNPs marked in bold were predicted binding site present in the predicted recognition cavity of the protein. B.S. stands for binding site

Interestingly, the presence of non-synonymous mutations correlated with change in bioactive conformation and drop in free energy (ΔG) of four proteins (g9986, g3683, g4104, g4641) belonging to three MFS and one recG (helicase) superfamilies in the test strain only (Fig. 2). The structural changes in these proteins lead to successful protein-ligand interactions.

## Discussion

As expected for functional conservation, majority of candidate genes in the TERIBR1 genome showed the presence of a large number of sSNPs and a few nsSNPs. Notably, 28/168 candidate genes contained only synonymous polymorphisms. Synonymous codon positions, though do not alter amino acid sequences of the encoded proteins, they may determine secondary structure, stability and translation rate of the mRNA [54]. Presence of sSNPs in the chromium tolerance candidate genes in the test strain could have affected folding and post-translational modifications of the nascent polypeptides which could in turn affect candidate protein expression and function towards Cr tolerance.

The polymorphism rate in 16 candidate genes that showed a high frequency of nsSNPs as compared to synonymous changes (Table 2) was ~ 16 SNPs/Kb with a frequency of ~ 11 nsSNPs/Kb. The observed high rate of nsSNPs in chromium-tolerance candidate genes of TERIBR1 as compared to the housekeeping genes (0.4 nsSNPs/kb; Table 4) could mirror environmental stress induced DNA variations and might provide an advantage in counteracting chromate stress. These included genes from mdrL/yfmO (12), recG (3) and chrE (1) families. The mdrL/yfmO genes belonged to the major facilitator superfamily (MFS), which codes for a metal ion-specific efflux protein [55]. High frequency of nsSNPs observed in the mdrL/yfmO genes in TERIBR1 could have led to altered protein structure and subsequent chromium efflux efficacy under extreme environmental condition, which we discussed in detail under the protein-ligand docking section. recG is a conserved enzyme present in bacteria, archaea, and eukaryota. *recG* encodes for the ATP-dependent recG DNA helicase which plays a critical role in DNA recombination and repair [56]. In vivo experiments conducted in *E. coli* showed that chromium salt stimulates several stress promoters associated with different types of DNA damage, indicating that DNA is one of the main targets for Cr (III) inside the cell [57]. After being internalized in cells Cr (VI) is reduced to Cr (III); recG eliminates polymerase arresting lesions (PALs), caused by Cr (III). The observed high frequency of nsSNPs in recG genes observed in our study might have resulted in higher efficiency of the enzyme to remove PAL lesions, thus mediating chromium stress tolerance in the fungal strain. In congruence, a study in *Pseudomonas corrugata* suggested that recG helicase played a crucial role in chromium tolerance by dismissing PAL lesions caused by Cr (VI)/Cr (III) [58]. The chrE gene encodes a rhodanese type enzyme [59]. Rhodanese protein subfamilies are suggested to be involved in different biological functions including cyanide detoxification, arsenic resistance and chromate responsive DNA-binding regulator. In addition, UniProt database defines ChrE as proteins involved in the processing of chromium-glutathione-complexes. An abundance of nsSNPs in these candidate genes for chromium tolerance could be the result of environment induced variations, perhaps for achieving functional relevance in TERIBR1. Environmentally guided changes in DNA and subsequently the proteins could be advantageous and may enable functional adaptation to extreme environmental influences [36].

**Table 3:** Docking analysis using PatchDock for selected proteins of *A. flavus* strain TERIBR1

Protein ID TERIBR1	Score	Area	ACE (kcal/mol)
g652	2764	330.5	−13.58
g9548	2496	304.5	−58.67
^b^g8975	2806	333.4	−29.90
g5755	2746	329.6	31.22
^b^g685	2576	321.4	−1.40
g641	2846	322.9	19.42
^b^g6212	2924	326.8	−62.95
^**a**^ **g9986**	**2644**	**296.6**	**−46.56**
g9401	2594	324.6	−77.47
^**a**^ **g3683**	**2788**	**306.3**	**−30.21**
g4359	2664	285.8	−60.63
^**a**^ **g4104**	**3034**	**335.4**	**−72.70**
^b^g9525	2966	362.2	−83.82
g712	2454	299.4	−7.63
g9088	2368	258	−62.84
^**a**^ **g4641**	**2772**	**302.9**	**−66.10**

^a^Protein - ligand interaction observed only in *A. flavus* strain TERIBR1

^b^Protein - ligand interaction observed in both the strains of *A. flavus*

The entries marked in bold indicate significant interaction of ligand with the protein

**Table 4:** SNP frequency in housekeeping genes in *A. flavus*

Gene ID NRRL3357	Gene ID TERIBR1	Annotation	Gene length (nucl)	change in nucl	change in aa	status of SNPs
AFL2T_10032	g899	Calmodulin	4047	0	0	0
AFL2T_10117	g962	RPL5 (ribosomal protein)	1071	0	0	0
AFL2T_03358	g2143	Polyketide Synthase Acetate	1692	0	0	0
AFL2T_03019	g2421	Chitin Synthase 1	2655	0	0	0
AFL2T_08232	g3003	cyclophilin	522	0	0	0
AFL2T_08160	g3065	Ubiquitin-conjugating enzyme	450	0	0	0
AFL2T_01340	g5399	Vacuolar protein sorting association protein	324	0	0	0
AFL2T_01191	g5533	Cytochrome oxidase	348	0	0	0
AFL2T_12005	g6415	Ubiquitin-conjugating enzyme	510	0	0	0
AFL2T_02547	g6955	Ubiquitin-conjugating enzyme	513	0	0	0
AFL2T_02762	g7132	L- Asparaginase	690	0	0	0
AFL2T_09767	g7323	ATP_D (ATP synthase subunit beta)	1821	0	0	0
AFL2T_06390	g8072	Polyketide Synthase Acetate	2529	0	0	0
AFL2T_09983	g10276	Ubiquitin-conjugating enzyme	501	0	0	0
AFL2T_09876	g10370	L- Asparaginase	1677	0	0	0
AFL2T_07389	g10698	Elongation Factor Alpha like protein	1185	0	0	0
AFL2T_06969	g3347	ATP_D (Atp synthase subunit beta)	1539	0	0	0
AFL2T_06937	g3377	Chitin Synthase 1	5283	0	0	0
AFL2T_05991	g6789	GAPDH/Glyceraldehyde 3-phosphate dehydrogenase	1524	0	0	0
AFL2T_02677	g7061	Vacuolar protein sorting association protein	1563	0	0	0
AFL2T_05240	g3927	cyclophilin	1122	0	0	0
AFL2T_02454	g5775	TBPI (tata box binding protein)	690	0	0	0
AFL2T_03769	g1786	Actin interacting protein 3	2931	0	0	0
AFL2T_05664	g6503	Histone	807	0	0	0
AFL2T_11201	g8361	Ubiquitin-conjugating enzyme	333	0	0	0
AFL2T_12447	g9127	Ubiquitin-conjugating enzyme	837	0	0	0
AFL2T_12048	g11077	DNA Topoisomerase II	1032	0	0	0
AFL2T_04711	g9681	Ubiquitin-conjugating enzyme	450	0	0	0
AFL2T_07021	g3301	Ubiquitin-conjugating enzyme	474	0	0	0
AFL2T_07052	g3271	Lactate Dehydrogenase A	1065	0	0	0
AFL2T_11998	g6422	Ubiquitin-conjugating enzyme	456	0	0	0
AFL2T_05713	g6542	Vacuolar protein sorting association protein	387	0	0	0
AFL2T_03033	g2409	Chitin Synthase 1	1671	0	0	0
AFL2T_08388	g2865	Vacuolar protein sorting association protein	2022	0	0	0
AFL2T_08078	g3130	Histone	429	0	0	0
AFL2T_05673	g6508	28 s rRNA	450	0	0	0
AFL2T_04621	g9757	cyclophilin	630	0	0	0
AFL2T_07907	g4829	Vacuolar protein sorting association protein	351	0	0	0
AFL2T_01105	g5607	Ubiquitin-conjugating enzyme	1167	0	0	0
AFL2T_09240	g6218	Ubiquitin-conjugating enzyme	558	0	0	0
AFL2T_09015	g9269	Polyketide Synthase Acetate	6828	0	0	0
AFL2T_10236	g1076	Vacuolar protein sorting association protein	2313	0	0	0
AFL2T_05795	g6613	28 s rRNA	1815	0	0	0
AFL2T_03329	g2169	Ubiquitin-conjugating enzyme	2631	0	0	0
AFL2T_09350	g6131	18 s rRNA	2382	0	0	0
AFL2T_00575	g419	Chitin Synthase 1	3588	C150T	H50H	sSNPs
AFL2T_00433	g530	Vacuolar protein sorting association protein	2562	C1483G	P496A	nsSNPs
AFL2T_02076	g8000	Elongation Factor Alpha like protein	3222	A890C	K297Q	nsSNPs
AFL2T_09781	g7310	Vacuolar protein sorting association protein	2925	T1645C	S548S	sSNPs
AFL2T_09150	g8774	Polyketide Synthase Acetate	7425	T5820C	L1938 L	sSNPs
AFL2T_06936	g3378	Chitin Synthase 1	5574	A315G, C2100T, A2841T	G105G, D700D, I947I	sSNPs
AFL2T_06204	g8236	Chitin Synthase 1	3315	C1668T	L556 L	sSNPs
AFL2T_02195	g6007	Vacuolar protein sorting association protein	4545	A2925G, C4044T	E975E, F1348F	sSNPs
AFL2T_08239	g2996	Calmodulin	5103	A260G, T2232C, C2757G	D87G, T744 T, L919 L	nsSNPs, sSNPs, sSNPs
AFL2T_07518	g5174	Polyketide Synthase Acetate	6366	G1575A, G3053A	S525 N, S1018 N	nsSNPs
AFL2T_00612	g388	ATP_D (Atp synthase subunit beta)	1671	A1515C	A505A	sSNPs
AFL2T_05167	g3861	Vacuolar protein sorting association protein	3528	G456 T, G807A	G152G, T269 T	sSNPs
AFL2T_04317	g9038	Vacuolar protein sorting association protein	1920	T1383C	I461I	sSNPs
AFL2T_12048	g6382	DNA Topoisomerase II	3183	T1871C	V624A	nsSNPs
AFL2T_12403	g9165	Polyketide Synthase Acetate	4944	T1265C, C2231T, Y2271G	M422 T, T744I, X757K	nsSNPs
AFL2T_08114	g3103	Elongation Factor Alpha like protein	1383	T273A	I91I	sSNPs
AFL2T_02416	g5810	Vacuolar protein sorting association protein	2124	C1606T	L536 L	sSNPs
AFL2T_06144	g8287	aflatoxin regulatory protein	945	C46T	L16F	nsSNPs
AFL2T_07648	g5058	Ubiquitin-conjugating enzyme	1278	T570C	G190G	sSNPs
AFL2T_03037	g2405	secretory lipase	1353	W1271A	X424N	nsSNPs
AFL2T_05603	g4255	Vacuolar protein sorting association protein	2757	C1164T, G1491 T, C1884T	I388I, T497 T, I628I	sSNPs
AFL2T_09157	g8767	Ras protein	6435	C1509T, A2193G, G2304A, T2358C, G3018C, G4056A	S503S, S731S, L768 L, N786 N, T1006 T, P1352P	sSNPs
AFL2T_12399	g9169	Chitin Synthase 1	3222	C1317G	V439 V	sSNPs
AFL2T_06989	g3329	Elongation Factor Alpha like protein	2583	T1461C	T487 T	sSNPs
AFL2T_11104	g8446	Polyketide Synthase Acetate	7482	T3962C, A5970G, A5981G	M1320 T, I1990M, D1994G	nsSNPs
AFL2T_01971	g7909	Vacuolar protein sorting association protein	5862	G281A, A2246T, A2316G, C2766T, T3135A	R94K, N749I, S772S, I922I, D1045E	nsSNPs, nsSNPs, sSNPs, sSNPs, nsSNPs
AFL2T_01302	g5433	Vacuolar protein sorting association protein	2457	C704T, T828C	A235V, G276G	nsSNPs
AFL2T_11645	g4481	ATP_D (Atp synthase subunit beta)	1116	T972C	I324I	sSNPs
AFL2T_04569	g9796	Elongation Factor Alpha like protein	2400	C546T, T1785C, T2253C	D182D, L595 L, F751F	sSNPs
AFL2T_05904	g6711	Elongation Factor Alpha like protein	2730	T146C, C1836T, G2002A, A2435G	F49S, P612P, V668I, D812G	nsSNPs, sSNPs, nsSNPs, nsSNPs
AFL2T_02030	g7958	Ubiquitin-conjugating enzyme	1176	T641C	V214A	nsSNPs
AFL2T_09952	g10303	TBPI (tata box binding protein)	1338	T789G	Y263Y	sSNPs
AFL2T_02696	g7079	Elongation Factor Alpha like protein	2169	T576C, A825T, G1332A, T1557C	D192D, I275I, E444E, F519F	sSNPs
AFL2T_12346	g8605	Elongation Factor Alpha like protein	2874	A1914G	E638E	sSNPs
AFL2T_10814	g1537	Ubiquitin-conjugating enzyme	708	C507T	D169D	sSNPs
AFL2T_07094	g3237	Polyketide Synthase Acetate	6606	T3478A, T4780C, G4927A, A5446G, G5677A, T5862C,T6264C	C1160S, Y1594H, E1643K, N1816D, A1893T, S1954S, C2088C	nsSNPs, nsSNPs, nsSNPs, nsSNPs, nsSNPs, sSNPs, sSNPs
AFL2T_02027	g7956	Vacuolar protein sorting association protein	1044	T663C	Y221Y	sSNPs
AFL2T_06635	g3639	L- Asparaginase	1074	A257G, C663A	D86G, N221 K	nsSNPs
AFL2T_09646	g7430	Ubiquitin-conjugating enzyme	921	T6C, G36C	S2S, A12A	sSNPs
AFL2T_01296	g5440	Vacuolar protein sorting association protein	876	C93T, G369A	T31 T, L123 L	sSNPs
AFL2T_05777	g6597	Vacuolar protein sorting association protein	2058	T538C, G1317A, G1438A, G1938C	Y180H, S439S, E480K, K646 N	nsSNPs, sSNPs, nsSNPs, nsSNPs
AFL2T_08606	g2683	Chitin Synthase 1	5172	G3339A, G3618A, A3831G, G4764C, G4952A	T1113 T, L1206 L, L1277 L, P1588P, R1651Q	sSNPs, sSNPs, sSNPs, sSNPs, nsSNPs
AFL2T_09101	g9340	Elongation Factor Alpha like protein	2859	C1173T, G1275A, T1895C, G1947A, A2172G, C2670T	D391D, L425 L, V632A, R649R, V724 V	sSNPs, sSNPs, nsSNPs, sSNPs, sSNPs
AFL2T_08131	g3088	cyclophilin	486	C158T	A53V	nsSNPs
AFL2T_00781	g238	Polyketide Synthase Acetate	6951	G2689A, C3072T, A3121G, A3864G	V897I, F1024F, F1041A, K1288 K	nsSNPs, sSNPs, nsSNPs, nsSNPs
AFL2T_01027	g12	Vacuolar protein sorting association protein	732	K401C	X134A	nsSNPs
AFL2T_00198	g739	Ubiquitin-conjugating enzyme	3240	C849A, T852A, G1475C, T1905C, A2459G, G2659A	F283 L, I284I, R492P, G635G, N820S, A887T	nsSNPs, sSNPs, nsSNPs, sSNPs, nsSNPs, nsSNPs
AFL2T_11313	g4750	aflatoxin regulatory protein	1164	T208C, C889A, G922A	S70P, E297E, G308R	nsSNPs, sSNPs, nsSNPs
AFL2T_08488	g2771	Elongation Factor Alpha like protein	3249	G1398A, A1683A, G2161A, G2328A	E466E, V561 V, A721T, Q776Q	sSNPs, sSNPs, nsSNPs, sSNPs
AFL2T_07094	g10842	Polyketide Synthase Acetate	1455	G526A, T711C, T1113C, A295G	A176T, S237S, L371 L	nsSNPs, sSNPs, sSNPs
AFL2T_06011	g6804	Ubiquitin-conjugating enzyme	504	A210G	P70P	sSNPs
AFL2T_01283	g5448	Chitin Synthase 1	2589	T1072C, A1536G	N358 N, K512 K	sSNPs
AFL2T_02416	g10910	Vacuolar protein sorting association protein	370	A55T	L18 L	sSNPs
AFL2T_05917	g6723	Ubiquitin-conjugating enzyme	741	A567G	K189 K	sSNPs
AFL2T_11034	g8506	GAPDH/Glyceraldehyde 3-phosphate dehydrogenase	1077	C237T, C357A	H79H, G119G	sSNPs
AFL2T_02787	g7154	Cytochrome oxidase	1482	A849G, C857T, A1003T,	E283E, T286I, T335F	sSNPs, nsSNPs, nsSNPs
AFL2T_03260	g2222	secretory lipase	1365	C936T, G985A, T990C, G1286A, T1291C	N312 N, G329R, T330 T, G429D, L431 L	sSNPs, nsSNPs, sSNPs, nsSNPs, sSNPs
AFL2T_07361	g10719	Lactate Dehydrogenase A	933	G702C, C753T, G879A	G234G, F251F, V293 V	sSNPs
AFL2T_09556	g7507	Ras protein	1458	C565T, G681A, T771C, T1047A	L189 L, T227 T, T257 T, P349P	sSNPs
AFL2T_03516	g2002	Vacuolar protein sorting association protein	2853	A1176C, C1180T	G392G, L394 L	sSNPs
AFL2T_04629	g9750	Elongation Factor Alpha like protein	1443	T475G, C543T, G1185C, T1302C	T181 T, V395 V, A434A	sSNPs
AFL2T_01738	g8814	cyclophilin	1638	A501C, A513G, A522G, A624G, T1011C, A1043G, T1176C	V167 V, E171E, V174 V, E208E, A337A, K348R, L392 L	sSNPs, sSNPs, sSNPs, sSNPs, sSNPs, nsSNPs, sSNPs
AFL2T_04801	g9604	Cytochrome oxidase	555	T207A, T463G	G69G, F155C	sSNPs, nsSNPs
AFL2T_12397	g9171	Vacuolar protein sorting association protein	1758	T1659C	G553G	sSNPs
AFL2T_08911	g9862	Polyketide Synthase Acetate	7170	T3097C, A3519C, G3689A, A3761T	W1033R, T1173 T, R1230Q, Y1254F	nsSNPs, sSNPs, nsSNPs, nsSNPs
AFL2T_00897	g134	cyclophilin	1893	G616 T, C855T, T1191C, A1222G	V206 L, F285F, Y397Y, T408A	nsSNPs, sSNPs, sSNPs, nsSNPs
AFL2T_04106	g10177	cyclophilin	642	A72T	T24 T	sSNPs
AFL2T_06925	g3387	Cytochrome oxidase	348	C63A	V21 V	sSNPs
AFL2T_07038	g3286	Chitin Synthase 1	2076	G1794A	T598 T	sSNPs
AFL2T_08473	g2782	cyclophilin	498	C320G	T107 T	sSNPs
AFL2T_01646	g2579	secretory lipase	909	C10T, T309C, G509C, C646T	L4L, H103H, R170P, L216 L	sSNPs, sSNPs, nsSNPs, sSNPs
AFL2T_03998	g10190	Histone	768	C96T, A255G, C391T	F32F, S85S, P131S	sSNPs, sSNPs, nsSNPs
AFL2T_07224	g5682	aflatoxin regulatory protein	1218	C318G, G408C, G552 T, C581T, A794G, A979G, G1075A, C1137T	T106 T, P136P, S184S, A194V, Y265C, S327G, V359 M, S379S	sSNPs, sSNPs, sSNPs, nsSNPs, nsSNPs, nsSNPs, nsSNPs, sSNPs
AFL2T_00797	g223	L- Asparaginase	1137	T426C, T693C, G831C, C855T, T858C	G142G, G231G, Q277H, I285I, D286D	sSNPs, sSNPs, nsSNPs, sSNPs, sSNPs
AFL2T_08030	g3169	secretory lipase	1269	A642G, A795G, T903C, A904G, A913G, T927C, T933C, T963C, C1140T	A214A, L265 L, Y301Y, N302D, I305V, D309D, F311F, N321 N, G380G	sSNPs, sSNPs, sSNPs, sSNPs, nsSNPs, sSNPs, sSNPs, sSNPs, sSNPs
AFL2T_08467	g2788	cyclophilin	1641	T83C, C408A, G610A, G655A, T666C, C690T, T750A, A789G	V28A, L136 L, A204T, A219T, F222F, Y230Y, T250 T, E263E	nsSNPs, sSNPs, nsSNPs, nsSNPs, sSNPs, sSNPs, sSNPs, sSNPs
AFL2T_04948	g9453	Polyketide Synthase Acetate	1977	C1314G, T1444C, G1519A, A1590G, C1767A, C1854T, G1962A	R438R, W482R, V507I, L530 L, I589I, N618 N, R654R	sSNPs, nsSNPs, nsSNPs, sSNPs, sSNPs, sSNPs, sSNPs
AFL2T_07791	g4925	Vacuolar protein sorting association protein	3813	G39A, C213G, G234A, A291G, C354T	L13 L, S71S, Q78Q, E97E, H118H	sSNPs
AFL2T_12205	g8728	secretory lipase	939	T25C, G182A, T380C, C435T, T699C, T714G, G768A, G828A	L8L, S61 N, I127T, S145S, I233I, L238 L, P256P, A276A	sSNPs, nsSNPs, nsSNPs, sSNPs, sSNPs, sSNPs, sSNPs, sSNPs
AFL2T_01987	g7921	cyclophilin	537	A504G	,K168 K	sSNPs
AFL2T_05263	g3947	Vacuolar protein sorting association protein	891	G301A, G401A, C558T, C654T, T749G, A750G, A775G	A101T, G134D, D186D, I218I, I250R, I250R, M259 V	nsSNPs, nsSNPs, sSNPs, sSNPs, nsSNPs, nsSNPs, nsSNPs
AFL2T_01745	g8808	GAPDH/Glyceraldehyde 3-phosphate dehydrogenase	1041	C192G, T222C, C345T, C348T, C393T, T474C	D64E, I74I,G115G, A116A, F113F, A158A	nsSNPs, sSNPs, sSNPs, sSNPs, sSNPs, sSNPs
AFL2T_04609	g9767	RPL5 (ribosomal protein)	531	C153T	Y51Y	sSNPs

Frequency of SNPs = 0.9 SNPs/kb

Frequency of sSNPs = 0.7 SNPs/kb

Frequency of nsSNP = 0.4 SNPs/kb

Several studies have shown that non-synonymous substitutions are likely to affect protein structure [60]. Mapping of nsSNPs to a known 3D structure reveals whether the replacement is likely to destroy the hydrophobic property of a protein, electrostatic interactions or interactions with ligands. Many nsSNPs have been found near or inside the protein-protein interaction interfaces that alter the protein function [61]. Sequence-based structure predictions help in identifying the positions of a protein that are located in the active site. Protein – ligand docking analysis further helps in identifying crucial amino-acids that are involved in ligand binding.

Non-synonymous mutations mediated change in free energy (ΔG) and concomitant bioactive conformation of four proteins (g9986, g3683, g4104, g4641) belonging to the MFS and recG helicase super families were noteworthy. A decrease in free energy and atomic contact energy (ACE) putatively resulted in target-ligand interaction with a significant PatchDock score in the case of the proteins coded by the chromium tolerant strain, TERIBR1 (Table 2); whereas no ligand interaction was observed in the corresponding proteins coded by reference strain. Figure 2 shows the results of the molecular docking studies of the four proteins (g9986, g4104, g4641, g3683) coded by TERIBR1 strain. Ligand binding free energy estimates (ACE) indicated a significant decrease in free energy of these proteins (Table 3). The nsSNPs in the candidate genes of the chromium tolerant *A. flavus* strain TERIBR1 seemed to have influenced protein structure that could have mediated protein and chromium interaction. However, not much overlapping between the predicted binding sites (by 3DLigandSite) and the ligand docking position was observed for these proteins. The multidrug transporters of the MFS superfamily are polyspecific and can extrude a remarkably diverse range of substrates. However, discussions pertaining to multi-substrate recognition and transport by members of the MFS are still open and it is not clear if the same amino acid residues are involved in substrate recognition and binding in varying conformations of the protein [62]. Biochemical studies on the *Escherichia coli* MFS drug/H+ antiporter concluded that the structural basis of substrate promiscuity is governed by a large, flexible and complex substrate recognition cavity within the protein, which enables different substrates to interact with different amino acid residues of the cavity, and to form different interactions with MFS transporter [63, 64]. The putative correlation between the influence of genetic polymorphisms on the structure and function of MFS transporters and chromium tolerance in *A. flavus* suggested the importance of efflux mechanism in microbial chromium tolerance. Our results supported previous reports of heavy metal efflux as one of the primary mechanisms of tolerance in microbial systems [65, 66]. Furthermore, ligand docking was observed in four proteins (g8975, g685, g6212, g9525) and their homologs coded by the test and the reference strains respectively. The non-synonymous amino acid changes in these cases seemed to have no influence on protein-ligand interaction.

In a recent study four populations of yeast, exposed to arsenic in its most toxic form, As (III), accumulated changes in DNA, adapted faster and went from poor to optimal performance for fitness components (length of lag phase, population doubling time and efficiency of growth) within just a few mitotic divisions. The study concluded that fitness component enhancements in yeast populations were adaptive responses to arsenic and not to other selective pressures [35]. The observed high rate of variations in the DNA of *A. flavus* strain TERIBR1 in our study, especially nsSNP polymorphisms, highlights the scope for additional research on genetic mechanisms operating in *A. flavus* in order to conclude on the role of stress mediated alterations in DNA on adaptation in micro-organisms.

## Conclusions

Changes in DNA, guided by extreme environmental conditions, could influence the structure of proteins important in chromium stress tolerance in *Aspergillus flavus*. The structural changes in transporter proteins and enzymes are expected to have potential influence on their functional efficacy. Our study provided insights into the genetic factors governing heavy metal tolerance, which may aid in the development of future heavy metal bio-remediation technologies. Further, to ensure that the genes presenting nsSNPs are involved in the tolerance to chromium of the TERIBR1 strain, the results obtained in the present study demand cross validation by a proteome analysis.

## Additional files

Additional file 1: **Table S1:** Representation of candidate genes for chromium tolerance in *A. flavus. (XLSX 97 kb)*

Additional file 2: **Table S2:** 16 genes coded by *A. flavus* strain TERIBR1 with high frequency of non-synonymous substitutions. (DOCX 15 kb)

Additional file 3: **Table S3:** Phyre2 prediction and analysis of secondary structure. (DOCX 18 kb)

Additional file 4: **Figure S1.** Protein-ligand interaction observed with homologous pairs of protein of *A. flavus* strains TERIBR1 and NRRL3357. (PDF 414 kb)

## Abbreviations

ACE: Atomic Contact Energy
BacMetdbs: Antibacterial Biocide & Metal Resistance Genes Database
CHR: Chromate Transport Protein
MFS: Major Facilitator Superfamily
NrBlast: Non Redundant Basic Local Alignment Search Tool
nsSNPs: Non Synonymous Single Nucleotide Polymorphisms
Phyre2: **P**rotein **H**omology/Analog**Y R**ecognition server
RCSB PDB: Research Collaboratory for Structural Bioinformatics Protein Data Bank
sSNPs: Synonymous Single Nucleotide Polymorphisms

## References

[1] Pérez Silva RM, Ábalos Rodríguez A, Gómez Montes De Oca JM, Cantero Moreno D (2009). Biosorption of chromium, copper, manganese and zinc by Pseudomonas aeruginosa AT18 isolated from a site contaminated with petroleum. Bioresour Technol.

[2] Shumate ES, Strandberg WG (1985). Accumulation of metals by microbial cells. Comprehensive Biotechnology.

[3] Andres Y, MacCordick HJ, Hubert JC (1992). Bacterial biosorption and retention of thorium and uranyl cations by mycobacterium smegmatis. J Radioanal Nucl Chem.

[4] Fourest E, Roux CJ (1992). Heavy metal biosorption by fungal mycilial by- products: mechanisms and influence of pH. Appl Microbiol Biotechnol.

[5] Hussein H, Krull R, SI AEL-ELA, Hempel DC (2001). Interaction of the different heavy metal ions with immobilized bacterial culture degrading xenobiotic wastewater compounds. Conference Proceedings.

[6] Hussein H, Farag S, Moawad H (2003). Isolation and characterisation of Pseudomonas resistant to heavy metals contaminants. Arab Journal of Biotechnology.

[7] Hansda A, Kumar V, Anshumali (2016). A comparative review towards potential of microbial cells for heavy metal removal with emphasis on biosorption and bioaccumulation. World J Microbiol Biotechnol..

[8] Lacina C, Germain G, Spiros NA (2003). Utilization of fungi for bio treatment of raw wastewaters. Afr J Biotechnol.

[9] Volesky B, Holan ZR (1995). Biosorption of heavy metals. Biotechnol. Progr..

[10] Huang CP, Huang CP (1996). Application of Aspergillus oryzae and Rhizopus oryzae. Wat. Res..

[11] Zafar S, Aqil F, Ahmad I (2007). Metal tolerance and biosorption potential of filamentous fungi isolated from metal contaminated agriculture soil. Biores Technol..

[12] Das S, Santra SC (2012). Bio-detoxification of chromium from industrial wastewater by fungal strains. BIOLOGIJA..

[13] Li ZH, Li P, Randak T (2011). Evaluating the toxicity of environmental concentrations of waterborne chromium (VI) to a model teleost, *Oncorhynchus mykiss*: A comparative study of in vivo and in vitro. Comp Biochem Physiol - C Toxicol Pharmacol.

[14] Bansal M, Singh D, Garg VK (2009). A comparative study for the removal of hexavalent chromium from aqueous solution by agriculture wastes’ carbons. J Hazard Mater.

[15] Baral A, Engelken RD (2002). Chromium-based regulations and greening in metal finishing industries in the USA. Environ Sci Pol.

[16] Viti C, Marchi E, Decorosi F, Giovannetti L (2014). Molecular mechanisms of Cr (VI) resistance in bacteria and fungi. FEMS Microbiol Rev.

[17] Levina A, Codd R, Dillon CT, Lay PA (2003). Chromium in Biology: Toxicology and Nutritional Aspects. Prog Inorg Chem.

[18] Samuel MS, Abigail ME, Ramalingam C (2015). Biosorption of Cr (VI) by Ceratocystis paradoxa MSR2 Using isotherm modelling, kinetic study and optimization of batch parameters using response surface methodology. PLoS One.

[19] Memon JR, Memon SQ, Bhanger MI, El-Turki A, Hallam KR, Allen GC (2009). Banana peel. A green and economical sorbent for the selective removal of Cr (VI) from industrial wastewater. Colloids Surfaces B Biointerfaces.

[20] Moussavi G, Mahmoudi M (2009). Removal of azo and anthraquinone reactive dyes from industrial wastewaters using MgO nanoparticles. J Hazard Mater.

[21] Anwar J, Shafique U, Waheed-uz-Zaman, Salman M, Dar A, Anwar S (2010). Removal of Pb (II) and Cd (II) from water by adsorption on peels of banana. Bioresour Technol.

[22] Gupta VK, Rastogi A, Nayak A (2010). Adsorption studies on the removal of hexavalent chromium from aqueous solution using a low cost fertilizer industry waste material. J Colloid Interface Sci.

[23] Wahab MA, Jellali S, Jedidi N (2010). Ammonium biosorption onto sawdust. FTIR analysis, kinetics and adsorption isotherms modeling. Bioresour Technol.

[24] Hamdi Karaoğlu M, Zor Ş, Uğurlu M (2010). Biosorption of Cr (III) from solutions using vineyard pruning waste. Chem Eng.

[25] Gutiérrez-Corona JF, Romo-Rodríguez P, Santos-Escobar F, Espino-Saldaña AE, Hernández-Escoto H (2016). Microbial interactions with chromium: basic biological processes and applications in environmental biotechnology. World J Microbiol Biotechnol.

[26] Cervantes C, Campos-García J, Devars S, Gutiérrez-Corona F, Loza-Tavera H, Torres-Guzmán JC, Moreno-Sánchez R (2001). Interactions of chromium with microorganisms and plants. FEMS Microbiol Rev.

[27] Srivastava S, Thakur IS (2006). Isolation and process parameter optimization of Aspergillus sp. for removal of chromium from tannery effluent. Bioresour. Technol..

[28] Gadd GM (1993). Interactions of fungi with toxic metals. New Phytol.

[29] Gadd GM, Mowll JL (1985). Copper uptake by yeast-like cells, hyphae and chlamydospores of Aureobasidium pullulans. Exp Mycol.

[30] Gadd GM (2009). Biosorption: critical review of scientific rationale, environmental importance and significance for pollution treatment. J Chem Technol Biotechnol.

[31] Shugaba A, Buba F (2012). Uptake and Reduction of Hexavalent Chromium by Aspergillus Niger and Aspergillus Parasiticus. J Phylogenetics Evol Biol.

[32] Pal C, Bengtsson-Palme J, Rensing C, Kristiansson E, Larsson DG (2014). BacMet: antibacterial biocide and metal resistance genes database. Nucleic Acids Res.

[33] Roberts RK, Marzluf AG (1971). The specific interaction of chromate with the dual sulfate permease systems of Neurospora crassa. Arch Biochem Biophys.

[34] Flores-Alvarez LJ, Corrales-Escobosa AR, Cortés-Penagos C, Martínez-Pacheco M, Wrobel-Zasada K, Wrobel-Kaczmarczyk K, Cervantes C, Gutiérrez-Corona F (2012). The Neurospora crassa chr-1 gene is up-regulated by chromate and its encoded CHR-1 protein causes chromate sensitivity and chromium accumulation. Curr Genet.

[35] Gjuvsland AB, Zörgö E, Samy JK, Stenberg S, Demirsoy IH, Roque F, Maciaszczyk Dziubinska E, Migocka M, Alonso Perez E, Zackrisson M, Wysocki R, Tamás MJ, Jonassen I, Omholt SW, Warringer J (2016). Disentangling genetic and epigenetic determinants of ultrafast adaptation. Mol Syst Biol.

[36] Richards EJ (2006). Inherited epigenetic variation-revisiting soft inheritance. Mycorrhiza.

[37] Sharma S, Adholeya A (2011). Detoxification and accumulation of chromium from tannery effluent and spent chrome effluent by Paecilomyces lilacinus fungi. Int Biodeterior Biodegrad.

[38] White TJ, Bruns TD, Lee SB, Taylor JW (1990). Amplification and direct sequencing of fungal ribosomal RNA Genes for phylogenetics. PCR - Protocols and Applications - A Laboratory Manual.

[39] Prasad P, Varshney D, Adholeya A (2015). Whole genome annotation and comparative genomic analyses of bio-control fungus Purpureocillium lilacinum. BMC Genomics.

[40] Allpaths LG: High quality genome assembly from low cost data:http://www.broadinstitute.org/software/allpaths-lg/blog/) Acceesed 9-oct-2016.

[41] MAKER: http://www.yandell-lab.org/software/maker.html. Accessed 20-oct-2016.

[42] InterProScan: protein sequence analysis & classification. (http://www.ebi.ac.uk/interpro/). Accessed 16-Nov-2016.

[43] Langmead B, Salzberg SL (2012). Fast gapped-read alignment with bowtie 2. Nat Methods.

[44] Kelley LA, Mezulis S, Yates CM, Wass MN, MJE S (2015). The Phyre2 web portal for protein modeling, prediction and analysis. Nat Protoc.

[45] Laskowski RA, MA MA, Moss DS, Thornton JM (1993). PROCHECK - a program to check the stereochemical quality of protein structures. J. App. Cryst..

[46] ERRAT: http://services.mbi.ucla.edu/ERRAT/ Accessed 10-Jan-2018.

[47] Guex N, Peitsch MC (1997). SWISS-MODEL and the Swiss-PdbViewer: an environment for comparative protein modeling. Electrophoresis.

[48] Wass MN, Kelley LA, MJE S (2010). 3DLigandSite: Predicting ligand-binding sites using similar structures. Nucleic Acids Res..

[49] Schneidman-Duhovny D, Inbar Y, Nussinov R, Wolfson HJ (2005). PatchDock and SymmDock: servers for rigid and symmetric docking. Nucleic Acids Res..

[50] Prabhu S, Rajeswari VD (2016). In silico docking analysis of bioactive compounds from Chinese medicine Jinqi Jiangtang tablet (JQJTT) using patch dock. J Chem Pharm Res.

[51] Berman J, Westbrook Z, Feng G, Gilliland TN, Bhat H, Weissig IN, PEB S (2000). The Protein Data Bank. Nucleic Acids Res.

[52] PyMOL Molecular Graphics System: https://www.pymol.org/. Accessed: 21-Jan-2018.

[53] Xue M, Yang J, Li Z, Hu S, Yao N, Dean RA, Zhao W, Shen M, Zhang H, Li C, Liu L, Cao L, Xu X, Xing Y, Hsiang T, Zhang Z, Xu JR, Peng YL (2012). Comparative analysis of the genomes of two field isolates of the rice blast fungus Magnaporthe oryzae. PLoS Genet..

[54] Shabalina SA, Spiridonov NA, Kashina A (2013). Sounds of silence: synonymous nucleotides as a key to biological regulation and complexity. Nucleic Acids Res.

[55] Romanova NA, Wolffs PF, Brovko LY, Griffiths MW (2006). Role of efflux pumps in adaptation and resistance of listeria monocytogenes to benzalkonium chloride. Appl Environ Microbiol.

[56] McGlynn P, Lloyd RG (2001). Rescue of stalled replication forks by RecG: simultaneous translocation on the leading and lagging strand templates supports an active DNA unwinding model of fork reversal and Holliday junction formation. Proc Natl Acad Sci U S A.

[57] Plaper A, Jenko-Brinovec S, Premzl A, Kos J, Raspor P (2002). Genotoxicity of trivalent chromium in bacterial cells: Possible effects on DNA topology. Chem Res Toxicol.

[58] Decorosi F, Tatti E, Mini A, Giovannetti L, Viti C (2009). Characterization of two genes involved in chromate resistance in a Cr (VI)-hyper-resistant bacterium. Extremophiles.

[59] Chihomvu P, Stegmann P, Pillay M (2015). Characterization and structure prediction of partial length protein sequences of pcoA, pcoR and chrB genes from heavy metal resistant bacteria from the Klip River, South Africa. Int J Mol Sci.

[60] Wu J, Jiang R (2013). Prediction of deleterious nonsynonymous single-nucleotide polymorphism for human diseases. Sci World J.

[61] Zhao N, Han JG, Shyu C-R, Korkin D (2014). Determining effects of non-synonymous SNPs on protein-protein interactions using supervised and semi-supervised learning. PLoS Comput Biol.

[62] Lewinson O, Adler J, Sigal N, Bibi E (2006). Promiscuity in multidrug recognition and transport: the bacterial MFS Mdr transporters. Mol Microbiol.

[63] Neyfakh AA (2002). Mystery of multidrug transporters: the answer can be simple. Mol Microbiol.

[64] Adler J, Bibi E (2004). Determinants of Substrate Recognition by the *Escherichia coli* Multidrug Transporter MdfA Identified on Both Sides of the Membrane. J Biol Chem.

[65] Ianeva OD (2009). Mechanisms of bacteria resistance to heavy metals. Mikrobiol.

[66] Kaur S, Kamli MR, Ali A (2011). Role of arsenic and its resistance in nature. Can J Microbiol.

