# Supplementary material for: Do environmentally induced DNA variations mediate adaptation in Aspergillus flavus exposed to chromium stress in tannery sludge?
Source: BMC Genomics. 2018 Dec 4;19:868. doi: 10.1186/s12864-018-5244-2 (PMC6278149; doi:10.1186/s12864-018-5244-2)
Supplement: Supplementary file 2 — Table S2: 16 genes coded by A. flavus strain TERIBR1 with high frequency of non-synonymous substitutions. (DOCX 15 kb) [file 12864_2018_5244_MOESM2_ESM.docx]

**Table S2: 16 genes coded by A. flavus strain TERIBR1 with high frequency of non-synonymous substitutions**

| **TERIBR1** | **Gene Family** | **Protein length (aa)** | **# nsSNP** |
| --- | --- | --- | --- |
| g652 | mdrL/yfmO | 417 | 3 |
| g9548 | mdrL/yfmO | 519 | 5 |
| g8975 | mdrL/yfmO | 613 | 5 |
| g5755 | mdrL/yfmO | 571 | 5 |
| g685 | recG | 816 | 5 |
| g6641 | mdrL/yfmO | 508 | 6 |
| g6212 | recG | 1353 | 7 |
| g9986 | mdrL/yfmO | 498 | 8 |
| g9401 | mdrL/yfmO | 656 | 8 |
| g3683 | recG | 908 | 7 |
| g4359 | chrE | 652 | 9 |
| g4104 | mdrL/yfmO | 443 | 10 |
| g9525 | mdrL/yfmO | 500 | 12 |
| g712 | mdrL/yfmO | 939 | 11 |
| g9088 | mdrL/yfmO | 308 | 13 |
| g4641 | mdrL/yfmO | 831 | 15 |
