# Supplementary material for: Do environmentally induced DNA variations mediate adaptation in Aspergillus flavus exposed to chromium stress in tannery sludge?
Source: BMC Genomics. 2018 Dec 4;19:868. doi: 10.1186/s12864-018-5244-2 (PMC6278149; doi:10.1186/s12864-018-5244-2)
Supplement: Supplementary file 3 — Table S3: Phyre2 prediction and analysis of secondary structure. (DOCX 18 kb) [file 12864_2018_5244_MOESM3_ESM.docx]

| **NRRL3357** | **TERIBR1** | **Template** | **Coverage** | **Identity** |
| --- | --- | --- | --- | --- |
| AFL2T_05698 | g652 | c1xzqA | 81% | 30% |
| AFL2T_04853 | g9548 | d1pw4a | 88% | 15% |
| AFL2T_04391 | g8975 | c4ldsB | 68% | 15% |
| AFL2T_02473 | g5755 | d1pw4a | 73% | 14% |
| AFL2T_00264 | g685 | c1z3iX | 55% | 27% |
| AFL2T_05826 | g6641 | d1pw4a | 83% | 13% |
| AFL2T_09247 | g6212 | c1z3iX | 55% | 27% |
| AFL2T_08767 | g9986 | d1pw4a | 86% | 13% |
| AFL2T_05032 | g9401 | c3ppsD | 34% | 30% |
| AFL2T_06586 | g3683 | c3mwyW | 72% | 27% |
| AFL2T_11779 | g4359 | c3utnX | 36% | 34% |
| AFL2T_09661 | g4104 | d1pw4a | 93% | 13% |
| AFL2T_04878 | g9525 | c4ldsB | 97% | 12% |
| AFL2T_00229 | g712 | c5ldgA | 29% | 23% |
| AFL2T_04255 | g9088 | d1pw4a | 78% | 11% |
| AFL2T_11442 | g4641 | d1pw4a | 73% | 13% |

**Table S3: Phyre2 prediction and analysis of secondary structure**

c1xzqA: Hydrolase probable trna modification gtpase trme; d1pw4a: MFS general substrate transporter; c4ldsB: responsible for bicyclomycin resistance; c1z3Ix: recombination/dna binding; [c3ppsD](http://www.sbg.bio.ic.ac.uk/phyre2/phyre2_output/b67b648c19e19aae/summary.html#c3ppsD_): oxidoreductase; [c3mwyW](http://www.sbg.bio.ic.ac.uk/phyre2/phyre2_output/a9935f5ee0f164e0/summary.html#c3mwyW_): hydrolase chromo domain-containing protein 1; [c3utnX](http://www.sbg.bio.ic.ac.uk/phyre2/phyre2_output/080d3b38aadd2058/summary.html#c3utnX_): transferase; [c5ldgA](http://www.sbg.bio.ic.ac.uk/phyre2/phyre2_output/42cad1e48b024491/summary.html#c5ldgA_): oxidoreductase;
