# Supplementary material for: Do environmentally induced DNA variations mediate adaptation in Aspergillus flavus exposed to chromium stress in tannery sludge?
Source: BMC Genomics. 2018 Dec 4;19:868. doi: 10.1186/s12864-018-5244-2 (PMC6278149; doi:10.1186/s12864-018-5244-2)
Supplement: Supplementary file 4 — Figure S1. Protein-ligand interaction observed with homologous pairs of protein of A. flavus strains TERIBR1 and NRRL3357. (PDF 414 kb) [file 12864_2018_5244_MOESM4_ESM.pdf]

Figure S1. Protein-ligand interaction observed with four homologous pairs of protein of *A. flavus* strains TERIBR1 and NRRL3357

(a)

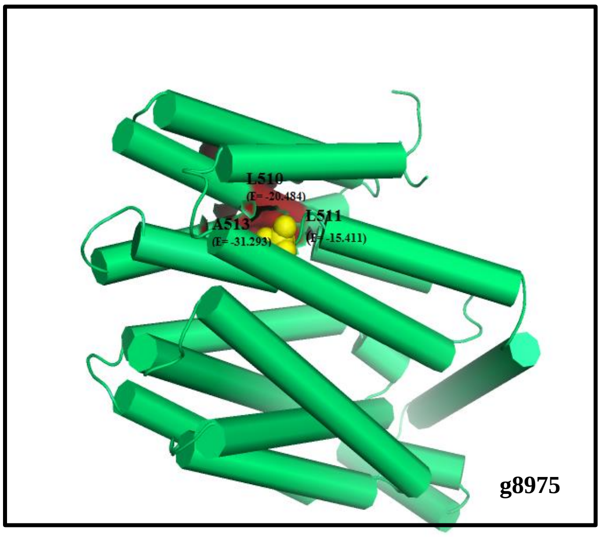

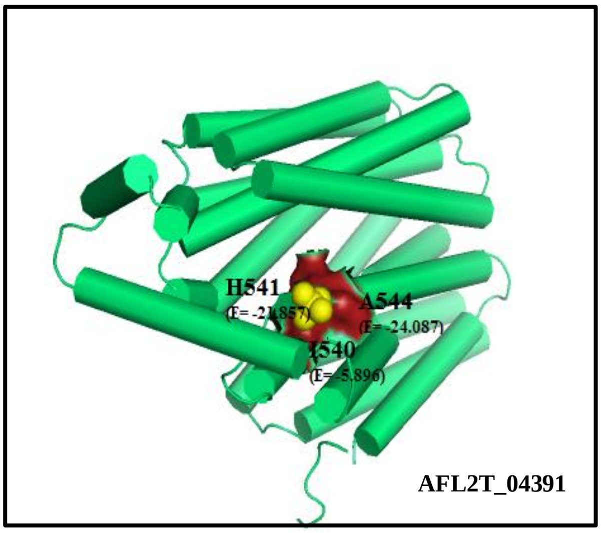

TERIBR1 **NRRL3357**

(b)

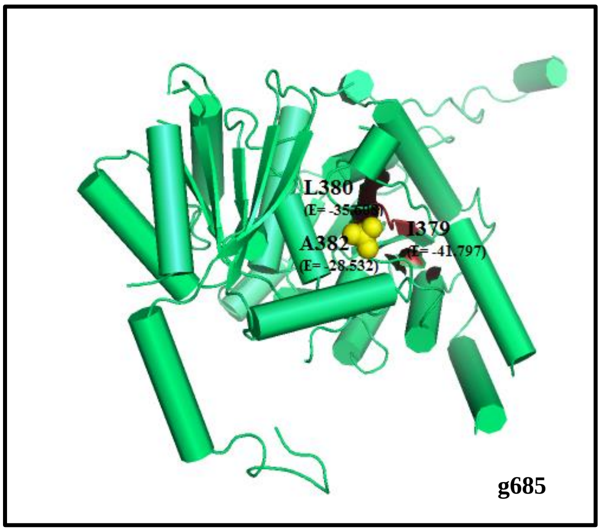

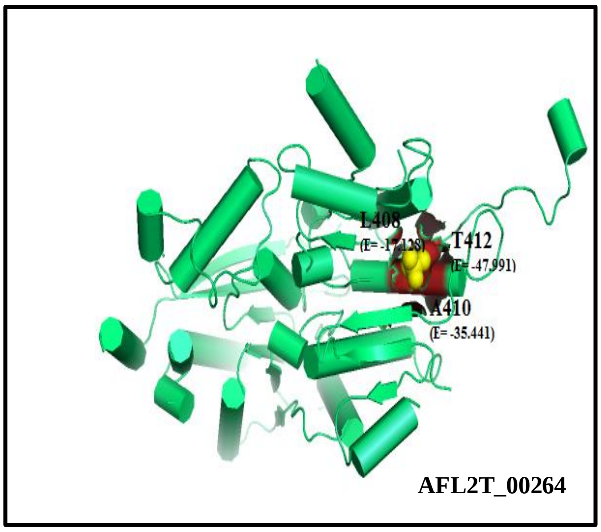

TERIBR1 **NRRL3357**

(c)

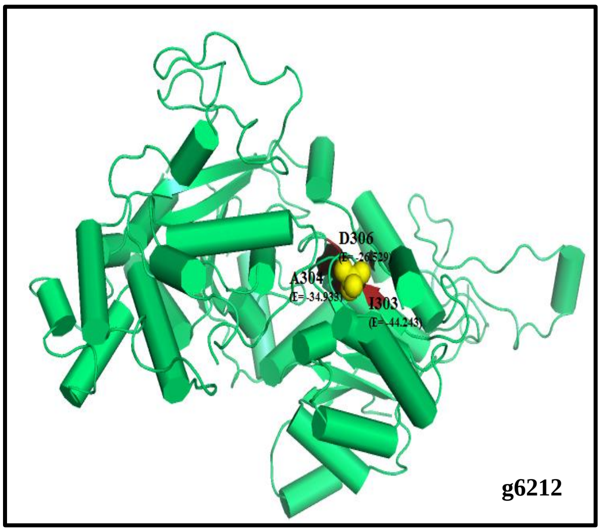

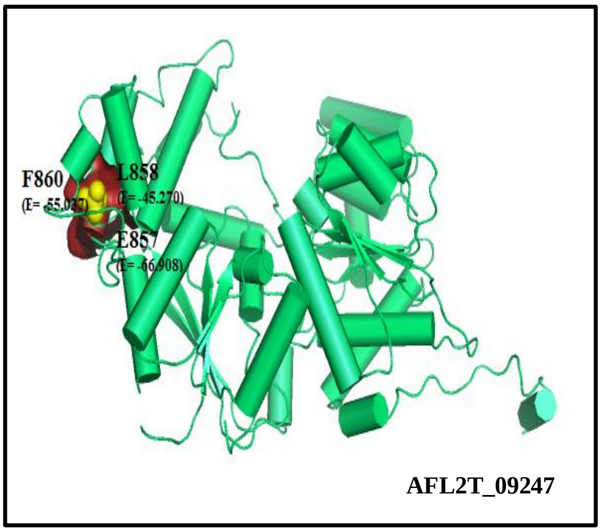

TERIBR1 **NRRL3357**

(d)

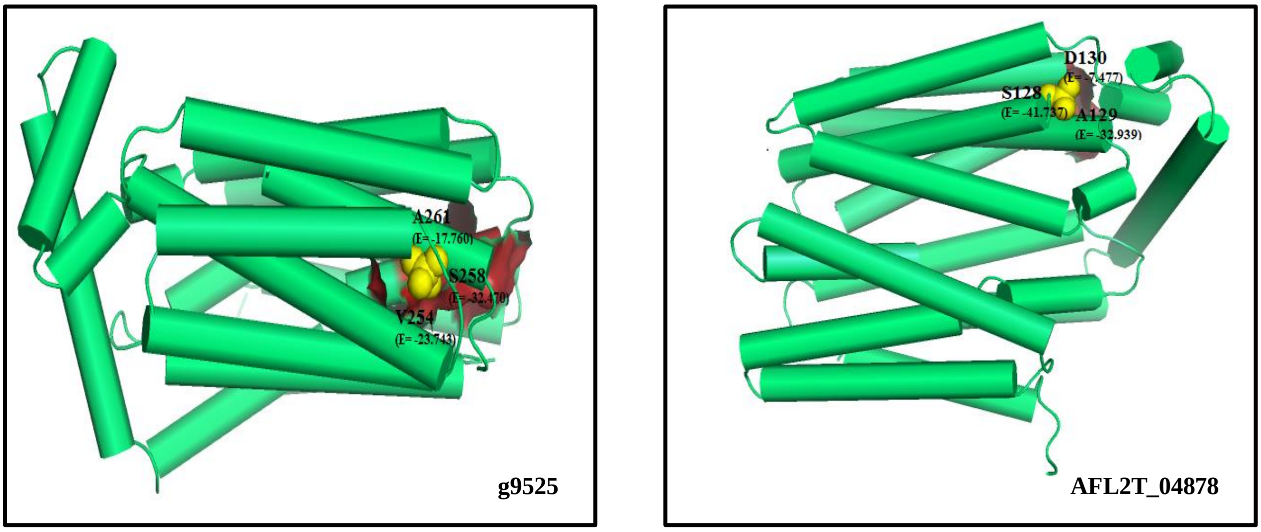

TERIBR1 NRRL3357

Chromate ion is depicted in yellow. Amino acid residues present in the proximity of ligand docking position are marked in black, binding sites predicted by 3DLigandSite are marked in fire brick and surfaced.
